# Personality and the Gender Gap in Self-Employment: A Multi-Nation Study

**DOI:** 10.1371/journal.pone.0103805

**Published:** 2014-08-04

**Authors:** Martin Obschonka, Eva Schmitt-Rodermund, Antonio Terracciano

**Affiliations:** 1 Center for Applied Developmental Science, Friedrich Schiller University, Jena, Germany; 2 Department of Psychology, Saarland University, Saarbrücken, Germany; 3 Department of Geriatrics, College of Medicine, Florida State University, Tallahassee, Florida, United States of America; University of Westminster, United Kingdom

## Abstract

What role does personality play in the pervasive gender gap in entrepreneurship across the globe? This two-study analysis focuses on self-employment in the working population and underlying gender differences in personality characteristics, thereby considering both single trait dimensions as well as a holistic, configural personality approach. Applying the five-factor model of personality, Study 1, our main study, investigates mediation models in the prediction of self-employment status utilizing self-reported personality data from large-scaled longitudinal datasets collected in the U.S., Germany, the U.K., and Australia (total *N* = 28,762). Study 2 analyzes (observer-rated) Big Five data collected in 51 cultures (total *N* = 12,156) to take a more global perspective and to explore the pancultural universality of gender differences in entrepreneurial personality characteristics. Across the four countries investigated in Study 1, none of the major five dimension of personality turned out as a consistent and robust mediator. In contrast, the holistic, configural approach yielded consistent and robust mediation results. Across the four countries, males scored higher on an entrepreneurship-prone personality profile, which in turn predicted self-employment status. These results suggest that gender differences in the intra-individual configuration of personality traits contribute to the gender gap in entrepreneurship across the globe. With the restriction of limited representativeness, the data from Study 2 suggest that the gender difference in the entrepreneurship-prone personality profile (males score higher) is widespread across many cultures, but may not exist in all. The results are discussed with an emphasis on implications for research and practice, which a particular focus on the need for more complex models that incorporate the role of personality.

## Introduction

Entrepreneurship (e.g., self-employment or venture creation) is more common among men than women and there is a lively debate how to explain this persisting and almost universal gender difference across the globe [1], [2], [3], [4]. This topic also received considerable attention by policymakers because it tackles the issue of gender inequality. Moreover, as entrepreneurship is often seen as driver of job creation and economic development, the gender gap in entrepreneurship may bring along a loss of benefits that would have been provided if more women engage in entrepreneurial activities. As stated in Kelley et al. [3], “when women do not participate equally in entrepreneurship, society loses out on the value that can be created by half its populace” (p. 5).

The present study contributes to this debate by a) looking at self-employment [5], [1], [6], [7], b) focusing on a “classical” topic in entrepreneurship research, namely personality differences [8], [9], [10], [11], and c) applying a global, cross-cultural perspective [3]. Consistent with Holland’s [12] general assumption that “the choice of a vocation is an expression of personality” (p. 7), recent research found clear support for the relevance of personality when investigating entrepreneurial career choice and behavior [10]. Accordingly, there is a renewed interest in entrepreneurship research in personality differences as an important determinant of entrepreneurial behavior, but the role of personality differences in the gender gap in entrepreneurial behavior is still poorly understood, particularly with regard to the five-factor model of personality (the Big Five: extraversion, conscientiousness, openness, agreeableness, and neuroticism [13]) and cross-cultural aspects. The present study thus analyzes data from different countries and cultures to examine whether gender differences in the individual personality make-up, assessed my means of the Big Five model, help to explain women’s lower propensity for self-employment (as well as the cross-cultural universality of this gender gap in entrepreneurial behavior).

Prior research on the role of personality in the gender gap in entrepreneurship has mainly investigated the mediating role of risk propensity and self-efficacy (e.g., [14], [15], [16]). The present study focuses on the five-factor model of personality [13], which is the more-established and cross-culturally validated model of personality [17]. Furthermore, this study differentiates between a variable-oriented and a person-oriented approach [18]. This strategy was inspired by recent findings indicating the relevance of looking at a global personality profile, in addition to the study of single traits in entrepreneurship research (e.g., [19]). In the present study, both the single Big Five traits (variable-oriented approach) and an entrepreneurship-prone Big Five profile (person-oriented approach) are studied as mediators between gender and self-employment status. Our primary analyses utilize large national studies from four countries, the U.S., Germany, the U.K., and Australia. The four samples use different self-report measures of the Big Five, which provide a test of the robustness of the associations across measures and nations. To apply a broader global perspective, this main analysis is complemented by an additional analysis of gender differences in observer-rated Big Five data from samples collected in a broader set of 51 cultures. This global perspective allows testing the pancultural universality/variability of gender differences.

### 1.1 The Gender Gap in Entrepreneurship and Personality: What We Know So Far

It is widely acknowledged that the gender gap in entrepreneurship is a complex and multi-causal phenomenon [3], [4]. The wide range of topics in this research field is remarkable, including demographic, psychological, sociological, and economic approaches. Past research focused, for example, on human capital and education [20], [21], social capital [3], gender-role stereotypes [22] as well as family responsibilities and other obstacles preventing women from pursuing entrepreneurship [3], [23].

In contrast, personality differences received much less attention, although it is well documented that, on average, men and women differ in their personality characteristics [24], [25], [26] **and that, at least to some extent, entrepreneurial behavior is the expression of personality [10].** What we know so far is that differences in risk propensity and self-efficacy play a role in gender differences in entrepreneurial career choice. Consistent with more general research on gender differences in risk propensity [27], and with research showing that entrepreneurs are less risk adverse than non-entrepreneurs [10], studies indicate that women show a lower propensity to engage in entrepreneurship because they are more risk adverse than men [28], [15], [29], [16], [30]. Concerning self-efficacy, other studies suggest that another reason for women’s lower propensity to engage in entrepreneurship is a lower confidence in their capabilities [31], [14], [4].

The question remains, however, which role basic personality characteristics play in the gender gap in entrepreneurship. The absence of empirical studies on this question is quite surprising, given that a) contemporary personality research is dominated by the five-factor model [32], b) there are reported robust gender differences in the Big five traits (e.g., women report higher levels of neuroticism, extraversion, agreeableness, and conscientiousness, [26]), c) existing biological and social psychological theories can explain these gender differences in the Big Five (see [24] for an overview of theories), d) the Big Five are known as important mediators in gender research [33], [34], and e) the Big Five predict entrepreneurial intentions and behaviors such as self-employed work (e.g., [6], [35], [36]), and channel a person’s occupational development from the early formative years in childhood and adolescence on towards career choices in adulthood, including becoming an entrepreneur [37], [38].

### 1.2 Assessing Personality: Looking at Single Traits vs. Trait Profiles

In personality psychology, two of the most commonly applied strategies to assess a person’s personality are the study of single traits (variable-oriented approach) vs. the study of intraindividual constellations of traits (person-oriented approach) [18]. The variable-oriented approach looks at isolated effects of single characteristics, whereas the person-oriented approach is interested in the person as a whole that functions as entity. Because both approaches have been successfully applied in prior entrepreneurship research (e.g., [37], [38], [36]), we decided to apply both approaches in the present study.

Consistent with a biological perspective on entrepreneurship [39], [40], existing studies at the level of single traits often found that entrepreneurship is related to higher levels of extraversion, conscientiousness, and openness, and to lower levels of agreeableness and neuroticism [35], [36]. These findings concur with findings from De Fruyt and Mervielde [41] who investigated Holland’s [12] RIASEC interest types and found that indicators of extraversion, conscientiousness, and openness were positively related with the enterprising type, whereas indicators of agreeableness and neuroticism had negative relationships (see also [42], [43]).

Complementing this variable-oriented research, studies could show that also a specific constellation of the Big Five traits within the person relates to entrepreneurship. Drawing from the variable-oriented findings on the single Big Five and the literature on entrepreneurial personality (e.g., [11]) and the enterprising type (e.g., [12]), an entrepreneurial personality profile is characterized by higher levels in extraversion, conscientiousness, and openness, and lower levels in agreeableness and neuroticism [37].

Ten years of research on such an entrepreneurship-prone Big Five profile delivered remarkably consistent results, indicating its validity in the prediction of entrepreneurial behavior as well as other entrepreneurial characteristics such as entrepreneurial human and social capital, intentions, self-identity, and cognitions (for the most recent overview of studies see [19]). This research also points to the direction that this personality profile is a more robust and consistent predictor of entrepreneurship and related variables than the single Big Five dimensions. Recent research further found that those regions in the U.S., Germany, and the U.K. where, on average, more people scoring high on the entrepreneurship-prone personality profile live, also have higher entrepreneurship rates (e.g., startup rates) [19]. In addition, these entrepreneurship rates in the region were particularly high if both came together in the region, a higher share of people scoring high on this personality profile and local business conditions conducive to entrepreneurship.

### 1.3 Aims of the Present Study

Our central aim was to test whether personality differences contribute to explain the gender gap in entrepreneurship. Study 1, our main study, thus investigates both the single Big Five and the entrepreneurship-prone Big Five profile as mediators between gender and self-employment. We analyze large national datasets to ensure considering the whole spectrum of personalities, occupations, and work settings in the real world. Another aim was to compare the mediation results across countries to check whether the single traits or the profile would deliver more consistent results [19]. The results from Study 1 are complemented by Study 2, which targets the generalizability of relevant gender differences in personality by focusing on a broader range of cultures and a different method of personality assessment.

## Study 1: Does Personality Help to Explain the Gender Gap in Self-Employment?

### 2.1 Method

In Study 1, we utilized data from four national longitudinal studies that a) were available to us and b) included reliable information on both the Big Five personality traits and self-employment status. These studies are the “Midlife Development in the U.S.” (MIDUS) study, the “German Socio-Economic Panel” (GSOEP), the “British Household Panel Study” (BHPS), and the “Household, Income and Labour Dynamics in Australia” (HILDA) survey. The Big Five data from these studies were already successfully utilized in a variety of studies, for example, in personality and developmental psychology (e.g., [44], [45], [46], [47], [48], [49], [50], [51]).

#### 2.1.1 Samples

The *Midlife Development in the U.S. (MIDUS)* study is a two-wave national study in the U.S. (Age of the respondents at Wave 1: 25–74 years). The study was conducted by the MacArthur Foundation Research Network on Successful Midlife Development. A first wave of data collection (T1) took place in 1995–1996, and a second in 2004–2006 (T2). More details on the background and design of this study are provided elsewhere [46], [52], [50]. In order to compare employed vs. self-employed individuals, we examined the 4946 participants who were working in the second wave.

The *German Socio-Economic Panel (GSOEP)* is a nationally representative longitudinal study of private households in Germany (for details, see [53]). Major topics of the annual surveys are household composition, occupation, employment, earnings, and health. The surveys are conducted by the German Institute for Economic Research (DIW), from which the data can be acquired. For the present analysis, we used data from the 2005 and 2006 waves and focused on people in work (either employed or self-employed in 2006) (*N* = 9,327).

The *British Household Panel Study (BHPS)*, which is carried out at the Institute for Social and Economic Research of the University of Essex, is a nationally representative longitudinal study of private households in the U.K. Major topics of the annual surveys are comparable with those in the GSOEP. Again, the present analysis analyzed data from the 2005 and 2006 waves and solely considered working individuals (*N* = 8,795).

The *Household, Income and Labour Dynamics in Australia (HILDA)* survey, which is managed by the Melbourne Institute of Applied Economic and Social Research, is a nationally representative longitudinal house-hold-based study in Australia. The topics of the survey overlap with those in the GSOEP and BHPS. Consistent with our German and British analyses, we utilized data from the 2005 and 2006 waves and considered people in work (*N* = 5,694).

#### 2.1.2 Measures. Gender

Gender was assessed by respondents’ self-reports [MIDUS: Wave 1, 0 = male (51.7%), 1 = female (48.3%); GSOEP: Wave 2005, 0 = male (55.1%), 1 = female (44.9%); BHPS: Wave 2005, 0 = male (51.3%), 1 = female (48.7%); HILDA: Wave 2005: 0 = male (46.6%), 1 = female (53.4%)].

**Big Five traits.** In the MIDUS, the Big Five traits were assessed by means of 25 adjectival items, selected from previous Big Five questionnaires (e.g., [54]). Examples of those adjectives are “moody” and “worrying” for neuroticism and “outgoing” and “lively” for extraversion. The response scale ranged from 1 (not at all) to 4 (a lot). As explained elsewhere (e.g., [46], [55], [50]), this measure has been validated, correlates with NEO traits scales, and shows high internal reliability coefficients. We analyzed Big Five data from Wave 1. Means were: extraversion: *M* = 3.20 (*SD* = 0.56); conscientiousness: *M* = 3.02 (*SD* = 0.53); openness: *M* = 3.42 (*SD* = 0.44); agreeableness: *M* = 3.49 (*SD* = 0.49); neuroticism: *M* = 2.34 (*SD* = 0.66).

In the GSOEP, a 15-item version of the Big Five Inventory (BFI, [54]) was used to assess the Big Five personality traits in the 2005 Wave (T1). Participants rated their personality characteristics using items such as “I am somebody who is shy” (seven-point Likert scales: 1 = does not apply at all, 7 = fully applies). A detailed description of the scale and evidence for reliability and validity in the SOEP data is provided in Gerlitz and Jürgen [56] and in Donnellan and Lucas [48]. The means of the single Big Five traits were: extraversion *M* = 4.89 (*SD* = 1.11), conscientiousness *M* = 6.03 (*SD* = 0.84), openness *M* = 4.56 (*SD* = 1.17), agreeableness *M* = 5.41 (*SD* = 0.97), and neuroticism *M* = 3.83 (*SD* = 1.20).

The same 15-item Big Five questionnaire used in the GSOEP was also employed in the 2005 Wave of the BHPS for the British respondents (see [45] for details on reliability and validity). The means of the single Big Five traits in the BHPS were: extraversion *M* = 4.58 (*SD* = 1.21), conscientiousness *M* = 5.40 (*SD* = 0.98), openness *M* = 4.59 (*SD* = 1.11), agreeableness *M* = 5.43 (*SD* = 0.96), and neuroticism M = 3.61 (*SD* = 1.24).

In HILDA, the Australian dataset, respondents completed a 36-item adjective-based measure of the Big Five that was developed from Saucier’s [57] Mini-Marker inventory. The response scale ranged from 1 (does not describe me at all) to 7 (describes me very well). Example adjectives are “orderly” and “systematic” for conscientiousness, and “creative” and “imaginative” for openness. Evidence for reliability and validity is provided in Cobb-Clark and Schurer [44] and Lucas and Donnellan [49]. The means of the traits in 2005 were: extraversion: *M* = 4.43 (*SD* = 1.07); conscientiousness: *M* = 5.10 (*SD* = 1.04); openness: *M* = 4.22 (*SD* = 1.06); agreeableness: *M* = 5.38 (*SD* = 0.93); neuroticism: *M* = 1.80 (*SD* = 1.09).

**Entrepreneurship-prone personality profile.** To quantify the profile, we applied the same fit-measure as in the previous research (e.g., [19], [58]). This fit-measure, which summarizes the single traits into one index, is comparable to Cronbach and Gleser’s [59] *D^2^*, which is a leading profile similarity measure in psychological research. By means of a fixed reference profile with extreme values as endpoints of the distributions (highest possible value in E, C, and O, lowest possible value in A and N) the individual deviation from these statistical endpoints is assessed for each person as *D^2^*. As discussed elsewhere [19], one must acknowledge that this is a relatively broadly defined measure (e.g., it does not look at the concrete shape of the empirical profile, for example if extraversion is higher than openness). There are no concrete theoretical and empirical reasons, however, for using other reference profiles. As only the individual empirical profile delivers variance in this assessment of the profile, whereas the reference profile is fixed for each person, such a fit-measure between a fixed reference profile and an empirical profile that delivers variance does not suffer from the common caveats associated with the comparison of two profiles that both deliver variance [60].

First, the original item scales were recoded from 1–4 to 0–3 in the MIDUS data, and from 1–7 into 0–6 in the GSOEP, BHPS, and HILDA data. Second, the goodness-of-fit between each person’s Big Five profile and the reference type, defined as the highest possible score (3 in MIDUS, 6 in GSOEP, BHPS, HILDA) in extraversion, conscientiousness, and openness, and the lowest possible score (0) in agreeableness and neuroticism, was calculated. To do this, each person’s squared differences between the reference values and their personal values on each of the five scales was defined. For instance, if a person scored 3 in neuroticism, the squared difference was 9 (because the reference value was 0). The five squared differences were then summed up for each person and the algebraic sign of this sum was reversed (e.g., a value of 20 became −20). The resulting value served as the final variable of the entrepreneurship-prone profile (MIDUS: *M* = −11.14, *SD* = 3.17; GSOEP: *M* = −44.46, *SD* = 14.07; BHPS: *M* = −46.57, *SD* = 14.15; HILDA *M* = −45.77, *SD* = 11.54). The higher this value (closer to zero), the higher the entrepreneurship-prone profile. Further details on this profile measure are provided in Obschonka et al. [19].

**Self-employment status.** To predict self-employment status, employed vs. self-employed individuals were compared. To achieve a chronological order between the personality measures and self-employment status, we used information about the employment status collected in the second wave in MIDUS, and in the year 2006 in the GSOEP, BHPS, and HILDA data. Of the wave 1 sample in MIDUS, 666 individuals were self-employed in wave 2, a percentage of 13.5%. Of the 2005 wave sample in the GSOEP, 965 individuals reported self-employment in 2006 (self-employed farmers and those helping in family businesses were not considered in this category, [61]), which amounts to a percentage of 10.3% self-employment in the entire sample of working individuals. The respective numbers were 1,100 self-employed (12.5% of the working population) in the BHPS, and 719 self-employed (11.5% of the working population) in the HILDA data. In the HILDA data, we had excluded cases that belonged to either the “employee of own business” category (6 percent) or to the “unpaid family workers” category (0.5 percent) because it was not clear to us whether the respective respondents were part of the group of employees or rather self-employed participants.

**Control variables.** Following earlier research on self-employment, which indicates that among sociodemographic variables, age and education (besides gender) are particularly relevant (e.g., [5], [61]), the present analyses were controlled for age and education. Prior research further indicates curvilinear effects (e.g., entrepreneurship may be more likely at both ends of the education distribution, [5]). We thus also controlled for curvilinear effects. In detail, we controlled for age and age^2^ in all four countries. To account for linear and curvilinear effects of education, in the GSOEP and in HILDA we used the length of education and length of education^2^ (years of education until Wave 2005). Such a variable on the actual length of education was not obtainable in the MIDUS and BHPS data so we used an education dummy from wave 1 in MIDUS and the 2005 wave in BHPS (0 = lower education, 1 = university degree), without considering a quadratic term (using length of education).

#### 2.1.3 Statistical Methods

In order to test for mediation effects [62], Preacher and Hayes’ [63] SPSS macro for assessing indirect effects in multiple mediator models were used. This macro estimates bootstrap confidence intervals for the indirect effect of each mediator in the mediation model. The indirect effects were estimated with 1,000 bootstrap resamples. The macro can deal with both continuous and binary dependent variables, which is an important feature because the outcome variable in our study is binary (employed vs. self-employed) (the macro uses logistic regressions in this case). Note that the following results (e.g., direct and indirect effects) refer to unstandardized regression coefficients (the macro does not estimate standardized coefficients). We used z-standardized values of the personality variables (the single Big Five traits and the Big Five profile) so that one can compare the results across the four countries.

### 2.2 Results

**Figure 1:**
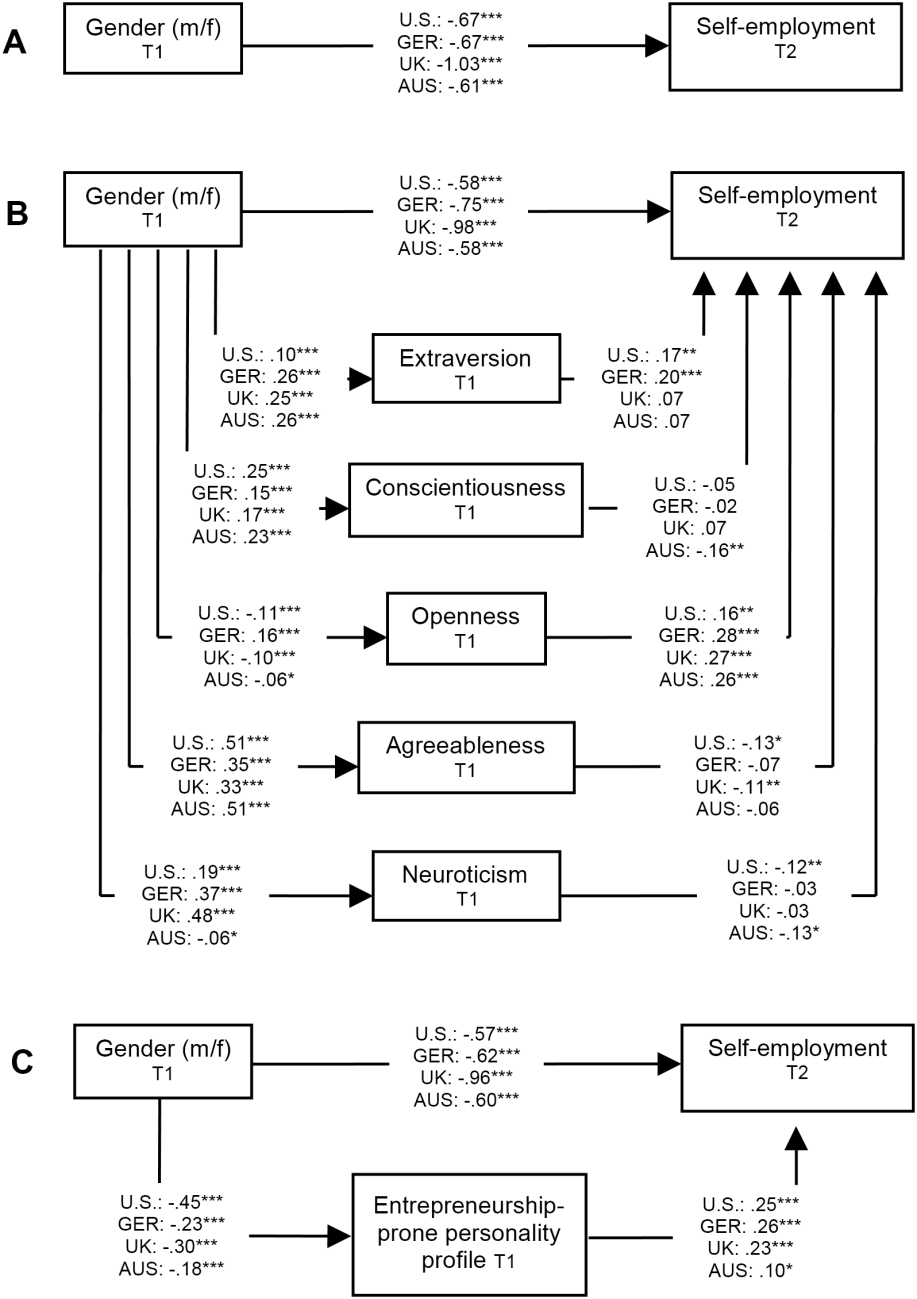
Mediation results across the four countries. A) Direct effect model, B) Mediation model with single Big Five traits as mediators, C) Mediation model with the entrepreneurship-prone personality profile as mediator. *Note*. All effects are controlled for age, age^2^, education, (and education^2^ in the German and Australian analysis). Unstandardized coefficients are given. All personality variables are z-standardized. U.S. = United States of America, GER = Germany, U.K. = United Kingdom. AUS = AUSTRALIA. *p<.05. **p<.01. ***p<.001.

In the following, we first present the mediation results for each country and then discuss their communalities and differences. The results from all four countries are illustrated in Figure 1.

#### 2.2.1 The “Midlife Development in the U.S.” (MIDUS) Study

As illustrated in Figure 1A, gender (0 = males, 1 = females) negatively predicted self-employment status in the U.S. (*B* = −.67, *SE* = .09, *p*<.001). Not surprisingly, women were self-employed less often. Applying a variable-oriented approach to capture personality by means of the Big Five, the single Big Five traits were introduced next as mediators into the model (Figure 1B). Gender positively predicted extraversion (*B* = .10, *SE* = .03, *p*<.001), conscientiousness (*B* = .25, *SE* = .03, *p*<.001), agreeableness (*B* = .51, *SE* = .03, *p*<.001), and neuroticism (*B* = .19, *SE* = .03, *p*<.001). Moreover, gender negatively predicted openness (*B* = −.11, *SE* = .03, *p*<.001). Extraversion (*B* = .17, *SE* = .06, *p*<.01) and openness (*B* = .16, *SE* = .06, *p*<.01) in turn had positive effects on self-employment status (0 = employed, 1 = self-employed), while agreeableness (*B* = −.13, *SE* = .05, *p*<.01) and neuroticism (*B* = −.12, *SE* = .05, *p*<.05) had negative effects. Conscientiousness showed no effect on self-employment (and could thus not mediate the gender–self-employment–link). The (unstandardized) indirect effect of gender on self-employment through extraversion was.02 (SE = .01; 95%CI = .01 and.04). The indirect effect of gender on self-employment through openness was −.01 (SE = .01; 95%CI = −.04 and −.01). The indirect effect of gender on self-employment through agreeableness was −.07 (SE = .03; 95%CI = −.12 and −.01). Finally, the indirect effect of gender on self-employment through neuroticism was −.02 (SE = .0195%CI = −.05 and −.01). This indicates significant mediation effects of openness, agreeableness, and neuroticism. Note that the indirect effect of extraversion is in the opposite direction (positive sign) than the direct effect to be explained (the negative effect between gender and self-employment), which speaks for a suppression effect, which is usually hard to interpret.

We then tested the entrepreneurship-prone Big Five profile, instead of the single traits, as mediator (Figure 1C). Gender negatively predicted the profile (*B* = −.45, *SE* = .03, *p*<.001), which in turn related positively to self-employment status (*B* = .25, *SE* = .05, *p*<.001). The indirect effect of gender through the profile was −.11 (SE = .02; 95%CI = −.17 and −.07). Hence, the entrepreneurship-prone personality profile mediated the gender-self-employment link. Given that there was still a (substantial) direct effect of gender on self-employment status in this mediation model, the profile did not fully but partially mediate the gender-self-employment link [62]. This does not come as surprise because, as stated earlier, a variety of additional factors should also play a role in the gender gap in entrepreneurship [4].

Note that the significant positive effect of the Big Five profile on self-employment also held when testing for males and females separately. This indicates that this profile matters in both genders alike.

#### 2.2.2 German Socio-Economic Panel (GSOEP)

As illustrated in Figure 1A, gender also predicted self-employment status in Germany (*B* = −.67, *SE* = .08, *p*<.001), with females reporting self-employment less frequently. Again, Gender positively related with extraversion (*B* = .26, *SE* = .02, *p*<.001), conscientiousness (*B* = .15, *SE* = .02, *p*<.001), openness (*B* = .16, *SE* = .02, *p*<.001), agreeableness (*B* = .35, *SE* = .02, *p*<.001), and neuroticism (*B* = .37, *SE* = .02, *p*<.001). Two of the Big Five traits showed effects on self-employment status, namely extraversion (*B* = .20, *SE* = .04, *p*<.001) and openness (*B* = .28, *SE* = .04, *p*<.001). The indirect effect of gender through extraversion on self-employment status was.05 (SE = .01; 95%CI = .03 and.08). The indirect effect of gender through openness on self-employment status was.04 (SE = .01; 95%CI = .03 and.06). As these indirect effects are both in the opposite direction than the direct effect, this does not indicate mediation but suppression.

When testing the entrepreneurship-prone Big Five profile instead of the single traits as mediator (Figure 1C), we again found a significant mediation effect of the profile. Gender negatively predicted the profile (*B* = −.23, *SE* = .02, *p*<.001), which in turn positively predicted self-employment status (*B* = .26, *SE* = .04, *p*<.001). As in the American analysis, this effect held true for males as well as for females. The indirect effect of gender through the entrepreneurial personality profile was −.06 (SE = .01; 95%CI = −.08 and −.04). This negative and significant indirect effect indicates mediation: The entrepreneurship-prone personality profile (partially) mediated the link between gender and self-employment status in the German data.

#### 2.2.3 British Household Panel Study (BHPS)

Gender also negatively predicted self-employment status in the British data (Figure 1A; *B* = −1.03, *SE* = .08, *p*<.001). Regarding mediation effects of the single Big Five traits we found the following (Figure 1B): Gender positively predicted extraversion (*B* = .25, *SE* = .02, *p*<.001), conscientiousness (*B* = .17, *SE* = .02, *p*<.001), agreeableness (*B* = .33, *SE* = .02, *p*<.001), and neuroticism (*B* = .48, *SE* = .02, *p*<.001). Gender had a negative effect on openness (*B* = −.10, *SE* = .02, *p*<.001). Two of the Big Five traits showed effects on self-employment status. Openness had a positive (*B* = .27, *SE* = .04, *p*<.001), and agreeableness a negative effect (*B* = −.11, *SE* = .04, *p*<.01). The indirect effect of gender on self-employment trough openness was −.03 (SE = .01; 95%CI = −.04 and −.02). Finally, the indirect effect of gender on self-employment through agreeableness was −.04 (SE = .01; 95%CI = −.07 and −.01). Hence, there was statistical indication for (partial) mediation effects of openness and agreeableness in the U.K.

Regarding the trait profile, we found the following (Figure 1C): Gender negatively predicted the entrepreneurship-prone profile (*B* = −.30, *SE* = .02, *p*<.001), which in turn positively predicted self-employment status (*B* = .23, *SE* = .05, *p*<.001). Again, this effect of the profile held true for males as well as for females. The indirect effect of gender through the profile was −.07 (SE = .01; 95%CI = −.10 and −.04), which indicates a significant (partial) mediation effect.

#### 2.2.4 Household, Income and Labour Dynamics in Australia (HILDA) Survey

In Australia, the gender effect on self-employment status was −.61 (*SE* = .08, *p*<.001, Figure 1A). As shown in Figure 1B, gender had a positive effect on extraversion (*B* = .26, *SE* = .03, *p*<.001), conscientiousness (*B* = .23, *SE* = .03, *p*<.001), and agreeableness (*B* = .51, *SE* = .02, *p*<.001), and a negative effect on openness (*B* = −.06, *SE* = .03, *p*<.05) and neuroticism (*B* = −.06, *SE* = .03, *p*<.05), respectively. Three Big Five traits predicted self-employment status: Openness had a positive (*B* = .26, *SE* = .05, *p*<.001), and conscientiousness (*B* = −.16, *SE* = .05, *p*<.01) and neuroticism (*B* = −.13, *SE* = .05, *p*<.05) a negative effect. The indirect effect of gender on self-employment trough conscientiousness was −.03 (SE = .01; 95%CI = −.06 and −.01). The indirect effect of gender on self-employment through openness was −.01 (SE = .01; 95%CI = −.03 and −.01). The indirect effect of gender on self-employment through neuroticism was.01 (SE = .01; 95%CI = .00 and.02). This indicates significant (partial) mediation effects of conscientiousness and openness, and a suppression effect of neuroticism.

Finally, gender negatively predicted the entrepreneurship-prone profile (*B* = −.18, *SE* = .03, *p*<.001), which in turn showed a positive effect on self-employment (*B* = .10, *SE* = .05, *p*<.05) (Figure 1C). Once again, this effect of the profile on self-employment status held in both genders. The indirect effect of gender through the entrepreneurial personality profile was −.02 (SE = .01; 95%CI = −.04 and −.00), indicating a significant (partial) mediation effect of the profile.

#### 2.2.5 Summary of Mediation Results across the Four Countries

When taking the results delivered by the national studies from the U.S., Germany, the U.K., and Australia together, a relatively clear overall picture emerged. Needless to say, females had a lower propensity of working self-employed than males in all four countries. This is the well-documented gender gap in entrepreneurship [3]. When comparing the coefficients across countries (Figure 1A), this gender gap was strongest in the U.K., while the other three countries show similar coefficients.

Regarding possible mediation effects of the *single Big Five*, the results are inconsistent and, in part, contradictory. While there was no mediation effects at all in the German analysis, openness turned out as valid mediator in the U.S., the U.K., and Australia (females were less open to new experiences than males; and a higher level in this openness was related to self-employment status). Moreover, agreeableness was a valid mediator in the U.S. and the U.K. (females were more agreeable; and lower values in agreeableness related to self-employment status). Neuroticism was a valid mediator in the American analysis only (females showed higher levels in neuroticism, these higher levels, in turn, were associated with a lower probability of self-employment). Finally, conscientiousness showed up as valid mediator in Australia only (females showed higher levels in this trait and such higher levels were associated with a lower propensity for self-employment).

These inconsistent variable-oriented results are in contrast to the results delivered by the *entrepreneurship-prone personality profile*. In all four countries under study the profile delivered a consistent picture: Males scored higher on this profile (the respective effect sizes were: U.S.: *d* = 0.50; Germany: *d* = 0.21; U.K.: *d* = 0.34; Australia: *d* = 0.21) and self-employment was more likely with higher values in the profile. This finding concurs with other studies indicating the relevance and robustness of this personality profile in entrepreneurship research, for example, when compared to the effect of the single Big Five dimensions and when comparing results across different samples und countries [19]. It is also consistent with seminal theorizing stressing that the entrepreneur is characterized by a combination of traits [11].

Our analyses had solely focused on working people. Is this gender difference in the entrepreneurship-prone profile restricted to people in work? Additional analyses revealed that the same gender gap (men scoring higher) was also present in the full 2005 GSOEP, BHPS, and HILDA waves and in the full MIDUS I wave as well as in those respondents that we had excluded for the purpose of the study (e.g., unemployed persons, homemakers).

## Study 2: Gender Differences in the Entrepreneurship-Prone Personality Profile across 51 Cultures

We then asked whether the observed gender difference in the entrepreneurship-prone Big Five profile in the four countries investigated in Study 1 (men score higher) a) extends to other countries and cultures across the globe, and b) can be replicated by means of observer-rated (instead of self-reported) personality data. Such questions on the pancultural universality of gender differences in personality have received considerable attention in cross-cultural research before (e.g., [24], [64], [26]), but never with regard to the entrepreneurship-prone Big Five profile.

### 3.1 Method

To consider a broader global perspective [3], we utilized (observer-rated) cross-cultural Big Five data from the Personality Profile of Culture Project [64], [65], [66]. This dataset has an overall sample size of *N* = 12,156 from 51 cultures (e.g., Brazil, China, Indonesia, Morocco, Peru, Russia, and Uganda). The respondents completed the observer-rating form of the Revised NEO Personality Inventory (NEO-PI-R), a 240-item questionnaire [67]. Students were instructed to rate the personality of a target person whom they knew well (a person living in the same culture/country of the observer). The data were recruited through a uniform sampling strategy across cultures mainly in the year of 2003, and are similar in size, age, and gender of the target persons that were rated by the observers. Therefore they lend themselves nicely for cross-cultural comparisons [64], [65], although one should keep in mind that these samples are not necessarily representative for the 51 cultures. The observer ratings substantially overlap with self-reported personality data [65], which also applies to cross-cultural gender differences in the Big Five traits [24], [64].

Using the raw scores of each respondent’s Big Five traits, we calculated the individual score of the entrepreneurship-prone Big Five profile for each target person (*N* = 12,156 altogether, for sample sizes per culture see Table 1) with a mean of −18.57 (*SD* = 4.06) in the overall dataset.

**Table 1:** Gender Difference in the Entrepreneurship-Prone Big Five Profile Across 51 Cultures.

Culture	*N (males/females)*	Direction of gender differences*(M* = *Males score higher; F* = *Females score higher)*	Significance of genderdifference	Effect size of genderdifference (*d*)
Argentina	104/100	F	ns	−0.14
Australia	105/101	M	ns	0.25
Austria	80/78	M	ns	0.15
Belgium	127/120	M	ns	0.23
Botswana	95/91	M	ns	0.13
Brazil	306/291	M	*p*<.05	0.18
Burkina Faso	102/105	M	*p*<.001	0.54
Canada	69/98	F	ns	−0.03
Chile	94/100	F	ns	−0.05
China	91/86	M	*p*<.05	0.31
Croatia	97/94	M	ns	0.01
Czech	170/230	F	ns	−0.04
Denmark	72/81	M	ns	0.21
England	95/99	M	*p*<.01	0.42
Estonia	160/138	M	ns	0.03
Ethiopia	99/98	M	ns	0.14
France	135/139	M	ns	0.05
Germany	156/437	M	ns	0.12
Hong Kong	105/102	M	*p*<.001	0.44
Iceland	99/100	M	ns	0.23
India	93/92	M	ns	0.25
Indonesia	97/99	M	*p*<.05	0.36
Iran	69/68	M	ns	0.02
Italy	96/99	M	ns	0.08
Japan	96/95	M	ns	0.23
Kuwait	218/250	M	*p*<.001	0.34
Lebanon	82/118	F	ns	−0.03
Malaysia	127/162	M	ns	0.22
Malta	101/101	M	*p*<.01	0.37
Mexico	93/80	M	ns	0.14
Morocco	98/73	F	ns	−0.06
N. Ireland	52/54	F	ns	−0.00
New Zealand	101/99	M	ns	0.06
Nigeria	90/94	F	ns	−0.04
Peru	67/87	M	*p*<.05	0.39
Philippines	99/98	M	ns	0.27
Poland	98/99	F	ns	−0.04
Portugal	101/97	M	ns	0.07
Puerto Rico	85/75	M	ns	0.01
Russia	154/166	F	ns	−0.14
S. Korea	101/95	F	ns	−0.08
Serbia	101/99	M	ns	0.18
Slovak	98/100	F	ns	−0.06
Slovenia	99/110	F	ns	−0.18
Spain	100/100	F	ns	−0.06
Swiss-French	128/137	M	*p*<.05	0.29
Swiss-German	98/116	M	ns	0.17
Thailand	106/103	M	ns	0.00
Turkey	104/104	M	ns	0.08
Uganda	83/83	M	ns	0.29
USA	456/463	M	ns	0.02

### 3.2 Results and Discussion

Using the software HLM [68], we employed multilevel modelling [participants (Level 1) nested within cultures (Level 2)] to estimate the fixed effect of gender on the personality profile across all 51 cultures. We also tested the heterogeneity of this effect in a random effects multilevel model (whether this effect differs significantly between cultures). The fixed effect of gender on the profile was *B* = −.047 (*SE* = 0.09; *p*<.001), which indicates that across the 51 cultures the average pattern is for women to score lower on this profile. Furthermore, both the intercept variance (0.91, *DF* = 50, *p*<.001) and the slope variance (0.14, *DF* = 50, *p*<.05) were significant, which means that there were significant differences across cultures in both the intercept and slope of the effect of gender on the personality profile.

In an additional analysis we estimated the gender difference in the personality profile separately for each of the 51 cultures. These results are summarized in Table 1, where we also report the sample sizes broken down by gender, and the direction, significance and effect size of the gender difference in the personality profile. In 37 of the 51 (72.5%) cultures, the direction of the gender difference concurred with the self-reported personality data from Study 1: Males showed higher values in the profile than females. Due to small sample sizes and low statistical power, however, this difference was significant in 10 cultures only. The effect sizes in these 37 cultures in which males had higher scores than females had a mean of 0.19 (*SD* = 0.14), with the highest effect in Burkina Faso (*d* = 0.54) and the lowest in Thailand (*d* = .00). Across the 14 cultures with higher absolute values for females than males in the personality profile, the effects were generally smaller than in the other cultures where males scored higher, and none of them reached the 5%-significance level. The effect sizes had a mean of 0.07 (*SD* = 0.05), with the highest effect in Slovenia (*d* = −0.18) and the lowest in Northern Ireland (*d* = −0.00).

Taken together, the results from Study 2 indicate that the gender difference in the entrepreneurial profile (men score higher) is indeed widespread across the globe, but may not exist in all cultures. Interestingly, the gender difference in the personality profile was not limited to Western countries, and there was not a clear demarcation between more or less developed countries [26]. It is thus somewhat difficult to answer the question of what could be the difference between the 37 cultures with a more entrepreneurial personality profile among males as opposed to the 14 cultures with no such difference or even a more entrepreneurship-prone personality profile among the females. There is no obvious geographical pattern. Maybe it is fair to say that the 14 are cultural groups that are characterized best by a quick change towards economic growth and development, a context often characterized by an upsurge of non-traditional, innovative views. Another interesting observation is that almost every single country of the former Eastern Bloc seems to appear in the list of the 14, cultures with no explicit tradition of entrepreneurship during their communist times. However, given that Canada was part of the list too, a country which does not match any one of the two categories, all interpretations of the lack of significant results have to remain purely speculative.

## General Discussion

This study makes a novel contribution to the current debate on the mechanisms behind the pervasive gender gap in entrepreneurship by focusing on self-employment status, the Big Five traits, and the pancultural universality/variability in relevant personality differences between the genders.

In Study 1, we did not find a consistent pattern for any of the five major dimensions of personality as mediators between gender and self-employment. The most consistent effects were found for Openness in three of the four countries. In contrast to these single trait results, the person-oriented analysis suggests that an entrepreneurial constellation of the Big Five traits within the person is a consistent mediator across the four countries. It should be noted at this point that, all in all, the respective mediation effects were rather small. This may have to do with the multicausality of the gender gap [3]. Besides personality, we already know that many other factors are playing a role for gender differences in entrepreneurial interests and activity (e.g., human and social capital, gender-role stereotypes, family responsibilities and so forth, [3]). Hence, the small effects are not very surprising. Psychological research demonstrated, for example, that if several factors – and not just one factor – affect a common outcome, the size of the effect of the single factors is often severely constrained [69]. This hints at the need for more complex explanation models of the gender gap in entrepreneurship. These models – and this is the basic message of the present study – should incorporate the role of personality, or as Rauch and Frese [10] put it: “we cannot develop a consistent theory about entrepreneurship if we do not take into consideration personality variables as well” (p. 61).

Study 2 provides first insights into the pancultural universality/variability of gender differences in the entrepreneurship-prone Big Five profile. We interpret these preliminary results as suggesting that this gender difference in entrepreneurial personality, which “favors” men, indeed shows a certain degree of pancultural universality. The results also suggest that the size of this gender difference in personality differs substantially across cultures; and that in some cultures this gender difference may not even exist, or may rather exist in the reverse direction – but we can only speculate here, given the small samples sizes.

### 4.1 Implications

Intervention programs aiming to stimulate entrepreneurship in more women by focusing on personality usually target “soft” personality traits like self-efficacy and risk taking, which can be successfully promoted via entrepreneurship education [70], [4]. In contrast, the Big Five are “hard” psychological characteristics that are less prone to contextual influence and relatively stable over time [13]. Taken our results with previous research on soft traits together, the picture of practical implications seems to be somewhat mixed when looking at the role of personality in the gender gap in entrepreneurship: On the one side, the documented link between soft personality characteristics and differences between men and women in entrepreneurship rates taps at possibilities to intervene, e.g., by promoting self-efficacy and risk taking in women. Besides other factors, the implementation of such promotion programs may have contributed to the observed increase in women entrepreneurship over the last decade. Wilson et al. [4], however, concluded that such “current trends mask the fact that men continue to be more active in entrepreneurship than women worldwide” (p. 387). Despite intensified efforts to substantially narrow this gap, the gender gap appears to be quiet robust across the globe. One possible driver of this (cross-cultural) persistence could be gender differences in the basic personality structure, as suggested by our analyses. For example, throughout the vocational development of men and women, gender differences in an entrepreneurial Big Five profile may be at work from early on, channeling women away from the world of entrepreneurship. Studies found that girls show less interest in an entrepreneurial career than boys [71]. Behind such career interests, in turn, may stand personality characteristics [43]. Indeed, past research found that those girls and boys, who scored higher on the entrepreneurial Big Five profile, as investigated in the present study, also had stronger entrepreneurial interests [37].

However, personality does not “mechanistically” determine one’s fate so that there should always be possibilities to effectively promote entrepreneurship also in those individuals scoring low in the entrepreneurial personality profile. Regarding their individual personality structure, women might have on average a lesser tendency, or attraction, towards an entrepreneurial career in many cultures across the globe, but this may not necessarily mean that they are per se less entrepreneurial or less talented for entrepreneurial activities than men. In fact, there are many successful female entrepreneurs and societies may want to intensify efforts to stimulate entrepreneurship in more women to finally narrow this gender gap and to utilize the full potential of female entrepreneurship.

Regarding implications for research, future studies on the role of personality in the gender gap in entrepreneurship should continue to consider a holistic approach (e.g., intraindividual trait patterns) in addition to the study of single traits. Both strategies (variable-oriented and person-oriented) may deliver important insights. It may not be enough to focus on soft traits only (e.g., self-efficacy), since broader and more stable traits also matter in that they canalize a person’s vocational development from childhood on [37]. Furthermore, future studies dealing with the gender gap in entrepreneurship could put a special focus on person-situation interactions as well as more complex models examining the interplay of (all) relevant factors driving this gender gap in the entrepreneurial behavior. Regarding the future study of the pancultural universality/variability of gender differences in personality characteristics relevant for entrepreneurship, the present results could inspire new research drawing from (more) representative cross-cultural samples, which might be available in the near future (e.g., within large-scale internet-based research projects, [72]).

### 4.2 Limitations

This study does not come without limitations. First, the outcome variable in our main analysis refers to self-employment, which is an often used proxy for entrepreneurship [5], [1], [6]. Many would agree, however, that business founding would be a more prototypical measure for entrepreneurship [3]. Such data on venture creation was not available in the four national datasets in Study 1. Prior research demonstrated, however, that the entrepreneurship-prone Big Five profile not only matters for self-employment but also for other entrepreneurial behaviors like business founding (e.g., [19], [58]).

Second, on the issue of assessment, it should be noted that in our primary analyses personality traits were assessed with short and different measures in the four samples. Therefore, some of the variability across samples may be due to the different coverage of each trait in each scale. It is remarkable, however, that the profile index produced consistent results despite such differences. Furthermore, an entrepreneur profile may be even better defined by accounting for the more specific personality facets that compose each Big Five factor. For example, among the components of extraversion, males tend to score higher on assertiveness, while females score higher on warmth. It is possible that a more in depth assessment of personality could advance current knowledge on the role of personality in entrepreneurship (e.g., by considering more specific personality facets or more fine-grained personality profiles).

Third, our mediation analyses are not controlled for other potential mediators as none of the factors in question were consistently available in the datasets (e.g., encouragement particularly for female self-employment). What was available, however, was a one-item measure of risk taking in the GSOEP (“Are you generally a person who is fully prepared to take risks or do you try to avoid risks?”, ten-point Likert scale; 0 = fully unwilling to take risks, 10 = fully willing to take risks). We controlled the indirect effects of gender on self-employment via the Big Five and the profile for the mediating effect of risk taking (risk taking was introduced as additional mediator in the models shown in Figure 1B and 1C). As a result, the mediation results concerning the single Big Five traits and the trait profile, as presented here, remained stable.

Fourth, we had investigated the effect of T1 personality on T2 self-employment status. Such an analysis, however, does not rule out the possibility that the effect could also operate the other way around, namely that entrepreneurial work shapes personality [73]. However, the Big Five are relatively stable over time [13] and prior research from a prospective developmental study found the entrepreneurship-prone Big Five profile, assessed as early as in adolescence, to predict entrepreneurial behavior over the subsequent occupational career in adulthood [38]. In an additional follow-up analysis with the (two-wave) American MIDUS data we tested whether the entrepreneurship-prone Big Five profile would also turn out as valid mediator between gender and T2 self-employment status when controlling for T1 self-employment status (around 10 years prior to T2). We again found a significant mediation effect. This also applied when comparing those who were not self-employed at T1 or T2 with those who worked as self-employed in T2 (those who started to work self-employed between T1 and T2). These additional analyses indicate that the mediating effect of the profile does not only hold in the prediction of self-employment *status* at a certain point in time but also in the prediction of *entry* into self-employment. Hence, all things considered, we have good reasons to assume that the Big Five structure affects entrepreneurial behavior in terms of a selection into a specific career, which is consistent with career choice theories giving personality a unique role [12], [74]. Socialization, the reversed effect with entrepreneurial behavior affecting the Big Five traits, on the other hand, could also be present, but may be weaker than the selection effect and could even act as a *promoter* of the relevant personality differences between males and females. As Roberts, Caspi and Moffitt [75] put it: “Work experiences […] make us more of who we already are” (p. 592). Thus, one could speculate that entrepreneurial work deepens and elaborates entrepreneurial characteristics. In this sense, it may also amplify gender differences in the entrepreneurship-prone personality profile. However, we have to stress that our data does not allow for strict causal conclusions.

Finally, the results from Study 2 should be generalized with caution, given the small and specific samples. Although these analyses make it possible to apply a global perspective, we see them as preliminary findings that complement our main analyses from another perspective. As stated elsewhere [64], since most raters where college aged, an adult perspective on personality might be lacking in these data. Moreover, there might be a certain “selection bias” affecting the results because the target persons could be chosen freely by the respondents. Finally, by means of these data we could not test whether the entrepreneurship-prone Big Five profile predicts self-employment in the cultures that were not considered in Study 1.

### 4.3 Conclusion

To conclude, the data from our main study suggest that not only the more “visible” gender differences such as those in human and social capital or situational obstacles preventing women from pursuing entrepreneurship (e.g., family responsibilities) matter in the gender gap in entrepreneurship, but also deeper factors that lie “under the skin”, something as fundamental as a person’s basic personality structure. The results thus illustrate the need for more complex, integrative models that a) consider personality, and its interplay with the other factors that together drive the gender gap in entrepreneurial behavior and b) inform the further development of promotion programs that stimulate female entrepreneurship more effectively. We also need more cross-cultural research to take into account the cultural context as well as potential cultural differences in the mechanisms behind the existing gender gap in entrepreneurship.
